# Pharmacological Inhibition of Protein Kinase C Reduces West Nile Virus Replication

**DOI:** 10.3390/v10020091

**Published:** 2018-02-23

**Authors:** Ana B. Blázquez, Ángela Vázquez-Calvo, Miguel A. Martín-Acebes, Juan-Carlos Saiz

**Affiliations:** Department of Biotechnology, INIA, Ctra. Coruña Km. 7.5, 28040 Madrid, Spain; angelavazquezcalvo@gmail.com (A.V.-C.); martin.mangel@inia.es (M.A.M.-A.); jcsaiz@inia.es (J.-C.S.)

**Keywords:** West Nile virus, viral replication, protein kinase C

## Abstract

Flaviviruses are relevant animal and human pathogens that include West Nile virus (WNV), Japanese encephalitis virus, dengue virus, or Zika virus, among others. Currently, no licensed therapy is available to fight flaviviral infections. Protein kinases C (PKCs) constitute a family of multifunctional lipid-dependent isoenzymes that regulate a wide variety of cellular processes (apoptosis, differentiation, proliferation, cellular transformation, motility, adhesion, etc.) being currently considered at the front line of drug development for the treatment of diverse human disorders. PKCs have also been implicated in different steps during viral replication; however, nowadays, results regarding their role in flavivirus replication are controversial. Here we demonstrate that calphostin C and chelerythrine, two broad-PKC inhibitors that target conventional, novel and atypical PKCs, significantly inhibit WNV multiplication in cell culture without affecting cell viability. A reduction of viral yields was observed in treated cells when compared with mock-treated cells. Likewise, immunofluorescence detection of viral enveloped E protein was reduced in treated cells, as was the amount of viral RNA released to the supernatant, mainly in those treated with chelerythrine. On the other hand, two PKC inhibitors specific for conventional and novel isoforms (staurosporine and enzastaurine) did not show any significant effect in WNV multiplication. These results suggested that PKCs, more probably atypical PKCs, are likely involved in WNV multiplication, although both broad-spectrum tested drugs seem to act through different mechanisms, and point to them as potential antiviral candidates for WNV, as well as for other related flaviviruses.

## 1. Introduction

West Nile virus (WNV) is a mosquito-borne pathogen responsible for recurrent outbreaks of febrile illness and encephalitis that belongs to the genus *Flavivirus* (family Flaviviridae). This genus includes other relevant human and animal pathogens, such as Japanese encephalitis virus (JEV), dengue virus (DENV), tick-borne encephalitis virus (TBEV), yellow fever virus (YFV), or Zika virus (ZIKV), among others. Flaviviruses are small (about 50 nm of diameter), spherical, enveloped RNA viruses, whose genomes are made up of a single-stranded molecule of positive polarity encoding three structural proteins (C, prM, and E) that participate in virion assembly, and seven non-structural proteins (NS1, NS2A, NS2B, NS3, NS4A, NS4B, and NS5) that play important roles in diverse functions during the flavivirus life cycle [1]. Non-structural proteins contribute to the establishment of the replication complex located in the endoplasmic reticulum (ER) membrane, modifying the architecture of the intracellular host membrane [2,3].

Protein kinases C (PKCs) constitute a family of multifunctional lipid-dependent isoenzymes that regulate a wide variety of cellular processes, like apoptosis, differentiation, proliferation, cellular transformation, motility, or adhesion [4,5,6]. Most PKC isoforms are ubiquitous and many cells co-express multiple PKC family members, however, some isoforms are expressed in a tissue-specific manner [7,8]. PKCs are divided in three subfamilies, depending on their sensitivity to activators, the requirement of a second messenger, and differences in the regulatory domain. Conventional PKCs (cPKCs: α, β and γ) require Ca^2+^, diacylglycerol (DAG), and a phospholipid to become activated; novel PKCs (nPKCs: δ, ε, η, θ, and µ) need DAG, but not Ca^2+^ for activation; and atypical PKCs (aPKCs: ι, λ, and ζ) require neither Ca^2+^ nor DAG [9]. PKCs are essential for melanoma progression and metastasis [10], and play important roles in the regulation of cell activation, proliferation [11], and differentiation [12]. PKC isoforms are also involved in different steps during viral replication, as reported for influenza virus [13], hepatitis E virus [14], human immunodeficiency virus type 1 (HIV-1) [15], or minute virus of mice (MVM) [16].

Apart from this role of PKCs in the control of cell biology, both in silico and in vitro analyses performed with DENV indicated that the flaviviral RNA-dependent RNA polymerase (NS5) contains four potential phosphorylation sites for PKC that are involved in the modulation of the viral replication. Three out of these four sites (Thr302, Ser796, and Ser885, according to DENV2 strain, Genbank AHG23127) are present in WNV, two of them (Thr302 and Ser796) being conserved in all vector-borne flaviviruses [17]. Phosphorylation of flavivirus NS5 has been widely reported [18,19,20,21,22]. Moreover, certain lipids required for the activation of atypical PKCs [23], such as ceramides, are increased in WNV-infected cells [24], pointing to a role of PKCs during WNV infection. However, previous studies with WNV and other flaviviruses reported conflicting results and did not evaluate the specific role of PKCs [17,25,26].

Considering that PKCs are at the front line of drug development for the treatment of diverse human disorders, such as tumorgenesis [10], diabetic complications [27], obesity [28], inflammation [29], and even viral infections [13,14,15,16], we have analyzed the effect of the inhibition of PKCs on WNV replication.

## 2. Materials and Methods

### 2.1. Cells and Viruses

Vero CCL81 (ATCC^®^ CCL-81^TM^) cells were grown in Eagle´s Minimum Essential Medium (EMEM; Lonza, Verviers, Belgium) containing 5% fetal bovine serum (FBS; Hyclone, GE Healthcare, Little Chalfont, UK), and supplemented with 2 mM l-glutamine and penicillin-streptomycin (Lonza, Verviers, Belgium), at 37 °C with 5% of CO_2_.

Vero cells were seeded in 24-well plates and monolayers (2 × 10^5^ cells/well) were infected with WNV New York/1999 (NY-99) strain (GenBank acc. n.: KC407666.1) for 1 h at 37 °C. Viral inoculum was then removed and replaced by fresh medium containing 2% fetal bovine serum. At 0, 8, 18, 24, 48, and 72 h post-infection (p.i.), supernatants were collected and frozen at −80 °C until used for infectious virus titer determination to generate viral growth curves.

Viral titers were determined on Vero cells by standard plaque assay in semisolid agarose medium as previously described [30]. A multiplicity of infection (MOI) of 0.5 plaque-forming units (PFU)/cell was used in all experiments.

### 2.2. Drug Treatments

Calphostin C (Merck, Burlington, MA, USA, https://pubchem.ncbi.nlm.nih.gov/compound/2533), chelerythrine chloride (Sigma, St. Louis, MO, USA, https://pubchem.ncbi.nlm.nih.gov/compound/2703), staurosporine (Selleckchem, Houston, TX, USA, https://pubchem.ncbi.nlm.nih.gov/compound/44259), and enzastaurin (Selleckchem, Houston, TX, USA, https://pubchem.ncbi.nlm.nih.gov/compound/176167) were used. Cells were infected, or mock-infected, and drugs were added when medium was replaced one hour after infection. Control cells were treated in parallel with the same amount of drug vehicle (DMSO). Drugs were tested at different concentrations (25 nM to 20 μM) and time points to find the most effective concentration of drug that exerted minimal effects on cell viability. In all cases, drugs were added to infected cultures 1 h p.i. to avoid possible interference with virus entry.

Lack of cellular toxicity of drug treatments was determined by measuring the cellular ATP content with CellTiter-Glo^®^ luminescent cell viability assay (Promega, Madison, WI, USA).

### 2.3. Quantitative RT-PCR

Viral RNA was extracted from culture supernatants using the Speedtools RNA virus extraction kit (Biotools B&M Labs, S.A., Madrid, Spain), and the amount of viral RNA copies was quantified by real-time RT-PCR [31]. The number of genomic equivalents to PFU/mL was calculated by comparison with a standard curve generated with previously-titrated viruses [32,33].

### 2.4. Antibodies

Mouse monoclonal antibody J2 against double-stranded RNA (dsRNA) (Scicons, Budapest, Hungary), rabbit polyclonal antibody (Thermo Scientific, Waltham, MA, USA), and mouse monoclonal antibody 3.67G (Millipore, Temecula, CA, USA), both directed against the E glycoprotein of WNV, and mouse monoclonal anti-β-actin (Sigma, St. Louis, MI, USA) were used as primary antibodies. TO-PRO-3, and secondary antibody against mouse IgG coupled to Alexa Fluor-488 were purchased from Life Technologies (Carlsbad, CA, USA). A secondary antibody against rabbit IgG (Dako, Santa Clara, CA, USA) and mouse IgG (Sigma, St. Louis, MI, USA) coupled to horseradish peroxidase were used in Western blot assays.

### 2.5. Immunofluorescence

Assays were carried out as previously described [34]. Briefly, cells were grown on glass cover slips, fixed with 4% paraformaldehyde in PBS (15 min room temperature), permeabilized with 1% BSA, 0.1% TritonX-100, 1M glycine in PBS for 15 min, incubated with primary antibody diluted in 1% BSA in PBS for 1 h, and then with fluorescently-conjugated secondary antibody (45 min). Finally, cells were incubated with TO-PRO-3 (5 min) and mounted with Fluoromount-G (SouthernBiotech, Birmingham, AL, USA). Samples were washed three times with PBS between each step. Cells were examined using a Leica TCS SPE confocal laser-scanning microscope (Leica, Wtezlar, Germany), and the images were acquired using Leica Advanced Fluorescence Software, and processed using ImageJ (http://rsbweb.nih.gov/ij/) and Adobe Photoshop CS2 (Adobe, San José, CA, USA).

### 2.6. Western Blot

Cells were lysed on ice in lysis buffer (150 mM NaCl, 5 mM β-mercaptoethanol, 1% NP-40, 0.1% sodium dodecyl sulfate (SDS), 50 mM Tris-HCl pH 8) supplemented with Benzonase Nuclease (Novagen, EMD Chemicals, San Diego, CA, USA) and cOmplete protease inhibitor cocktail tablets (Roche, Indiannapolis, IN, USA). Protein concentration was determined by Bradford assay. Equal amounts of proteins were mixed with Laemmli sample buffer, subjected to SDS-PAGE, electrotransferred onto polyvinylidene fluoride (PVDF) membranes, and blocked with 5% skimmed milk in PBS 0.05% Tween-20. Then, membranes were incubated overnight at 4 °C with primary antibodies, washed three times with PBS-Tween, and subsequently incubated for 1 h at room temperature (RT) with secondary antibodies coupled to horseradish peroxidase diluted in 1% skimmed milk in PBS-Tween. Next, membranes were washed three times with PBS-Tween and proteins were detected by chemiluminiscence using a ChemiDocTM XRS+ System (Bio-Rad, Hercules, CA, USA).

### 2.7. Statistical Analyses

To test the significance of the differences, one-way analysis of the variance (ANOVA) was performed using the statistical package SPSS 15 (SPSS Inc, Chicago, IL, USA), applying Bonferroni’s correction for multiple comparisons. Data are presented as the mean ± standard deviation. Asterisks denote statistically significant differences at *p* < 0.05.

## 3. Results

### 3.1. Inhibition of WNV Multiplication after Treatment with PKC Inhibitors

**Figure 1:**
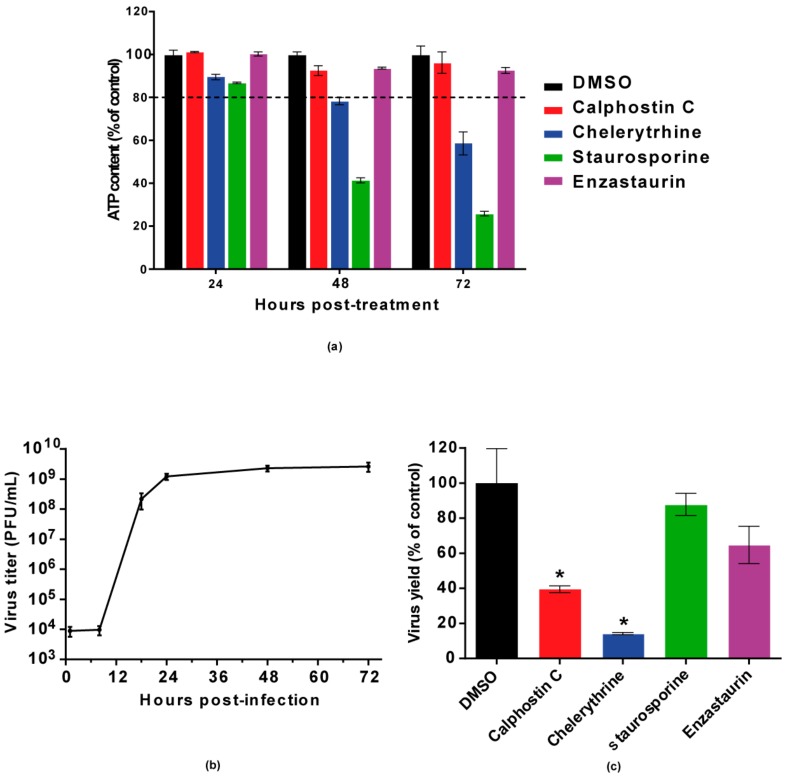
Inhibition of WNV multiplication in Vero cells treated with PKC inhibitors. (**a**) Cell viability was determined as ATP content in mock-infected cells treated with the drugs (200 nM calphostin C, 8 µM chelerythrine, 200 nM staurosporine, and 200 nM enzastaurin) at 24, 48, and 72 h. The dotted line indicates 80% of the viability of control cells; (**b**) A full viral growth was performed in untreated infected cells (multiplicity of infection (MOI) of 0.5 PFU/cell) by determining virus yields in supernatants collected at 1, 8, 18, 24, 48, and 72 h p.i.; (**c**) Cells were infected with an MOI of 0.5 PFU/cell, and treated with the drugs at the concentration used in (**a**) as described in the Materials and Methods section. Then, virus yields in culture supernatants were determined by plaque assay at 24 h p.i. Statistically significant differences are denoted as * *p* < 0.05. Figures represent the average of three independent experiments.

**Figure 2:**
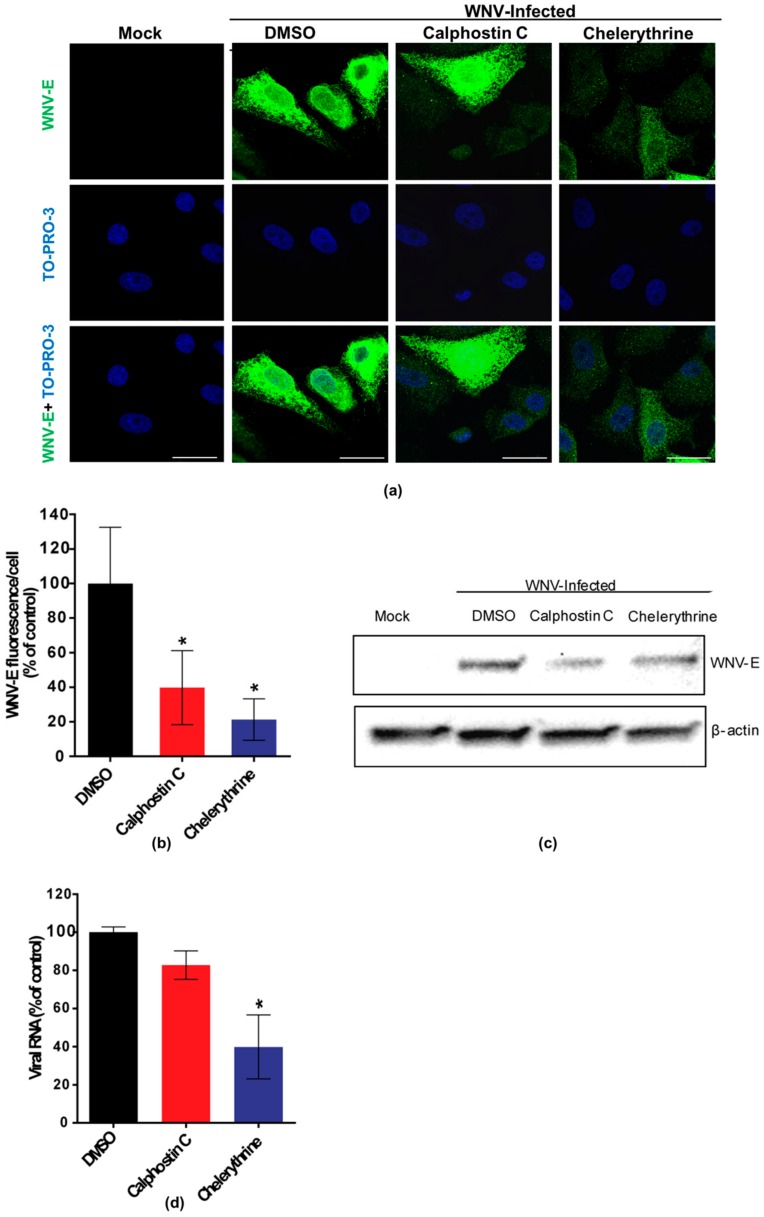
Visualization of the expression of WNV E glycoprotein in cells treated with PKC inhibitors. (**a**) Cells were infected with an MOI of 0.5 PFU/cell, treated with 200 nM calphostin C or 8 µM chelerythrine, fixed at 24 h p.i., and processed for immunofluorescence using a monoclonal antibody against WNV-E protein and a secondary antibody coupled to Alexa Fluor 488. Nuclei were stained with TO-PRO 3. Mock-infected cells were processed in parallel and included as control. Scale bars: 25 µm; (**b**) Quantification of the fluorescence intensity of anti-E antibody in cells infected and treated or not with drugs; (**c**) Vero cells were infected with a MOI of 0.5 PFU/cell and treated with calphostin C or chelerythrine as in (**a**). Cells were lysed and WNV E glycoprotein was detected by Western blotting using a polyclonal specific antibody. Membrane was reincubated with an anti-β-actin antibody as a control for protein loading; (**d**) Reduction of WNV RNA in the supernatants from cells treated with the PKC inhibitors. Vero cells were infected with an MOI of 0.5 PFU/cell and treated with the drugs, as in (**a**). Culture supernatants were collected at 24 h p.i., and the amount of viral RNA was determined by quantitative RT-PCR. Statistically significant differences are denoted as * *p* < 0.05. Figures represent the average of three independent experiments.

Calphostin C and chelerythrine, broad-PKC inhibitors with different sites of interaction within the PKC protein structure, and staurosporine and enzastaurin, specific inhibitors for conventional and novel PKC isoforms [35,36], were tested for their capability to inhibit WNV multiplication in Vero cells. Calphostin C, with reported IC_50_ of 50 nM (https://pubchem.ncbi.nlm.nih.gov/compound/2533), and chelerythrine, with IC_50_ of 0.66 µM [37], were used at 200 nM and 8 µM, respectively. Staurosporine, with IC_50_ of 2,7 nM [38] and enzastaurin, with IC_50_ of 2–73 nM [35], were both used at 200 nM. In order to determine the optimal point to analyze the effect of the drugs on WNV infection, cell viability was assayed at different time points from 24 to 72 h after drug treatment (Figure 1a) and a viral growth curve was determined (Figure 1b). Considering these results, samples were analyzed at 24 h p.i., when viral yields were at the plateau (Figure 1b) without drugs affecting cell viability (higher than 80%) (Figure 1a). At the tested concentrations, only calphostin C and chelerythrine significantly inhibited WNV multiplication (Figure 1c). No effect was observed neither with staurosporine nor with enzastaurin, which inhibit both conventional and novel PKC isoforms (Figure 1c). Detection of the WNV envelope (E) glycoprotein in virus-infected cells treated, or not, with the drugs that exerted a previous inhibitory effect was performed by immunofluorescence and Western blot, confirming these results. The intensity of fluorescence was significantly reduced in infected cells treated with either drug when compared to non-treated control infected cells (Figure 2a,b), as was the WNV-E protein expected band observed by Western blot (Figure 2c). However, while quantitative RT-PCR analysis of the amount of viral RNA released to the supernatant 24 h p.i. was significantly reduced in infected cells treated with chelerythrine, an almost negligible decrease was observed in calphostin C treated cells (Figure 2d). Taken together, these results suggested that PKCs, mainly those of the atypical subfamily, are likely involved in WNV multiplication, even though differences in the specificity for PKC isoforms displayed by drugs produced differences in their inhibitory effect.

### 3.2. Chelerythrine Inhibition Reduces WNV Infection at a Replication Step

**Figure 3:**
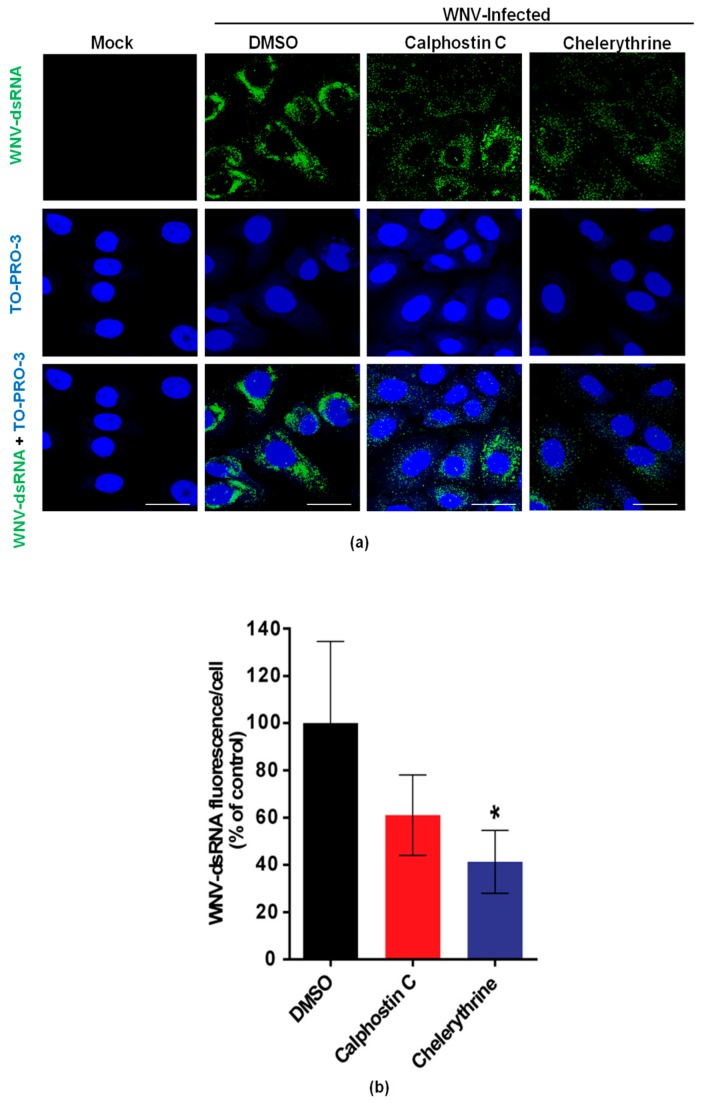
Visualization of intracellular dsRNA accumulation in infected cells treated with PKC inhibitors. (**a**) Cells were infected with an MOI of 0.5 PFU/cell, treated with the drugs as in Figure 1, fixed at 24 h p.i., and processed for immunofluorescence using a monoclonal antibody against dsRNA and a secondary antibody coupled to Alexa Fluor 488. Nuclei were stained with TO-PRO 3. Mock-infected cells were processed in parallel and included as control; Scale bar: 25 µm (**b**) Quantification of the fluorescence intensity of dsRNA in cells infected and treated or not with drugs as shown in (**a**). Statistically significant differences are denoted as * *p* < 0.05. Figures represent the average of three independent experiments.

To evaluate the effects of calphostin C and chelerythrine on the development of WNV-replication complexes, the amount of dsRNA intermediates, a widely-used maker of flavivirus replication complex, was assessed by immunofluorescence in WNV-infected cells treated, or not, with the drugs. As expected, no dsRNA was detected in mock-infected cells, whereas it was observed in infected cells (Figure 3a). A reduction of the fluorescence intensity was observed in infected cells treated with either drug (Figure 3b), being significantly lower in those treated with chelerythrine, thus, further confirming the impairment in WNV viral replication exerted by this PKC inhibitor.

## 4. Discussion

As currently no antiviral therapies against any flaviviruses, including WNV, have been approved, the search for inhibitory compounds is a keystone for therapeutic applications [39,40,41]. In this work, the effect of two broadly-active PKC inhibitors, calphostin C, a potent inhibitor used in the treatment of some cancer cell lines [42,43], and chelerythrine, with broad-spectrum biological activities, such as antimicrobial, antifungal, anti-inflammatory, and anti-tumoral effects [44], and two PKC inhibitors that have shown specific activity against conventional and novel PKC isoforms, sotrastaurine and enzastaurin, have been assayed against WNV infection in cell culture. Our results showed that both wide-spectrum PKC inhibitors (calphostin C and chelerythrine) induced a statistically significant reduction in WNV multiplication, while no effect was observed neither with sotrastaurine, nor with enzastaurin. Moreover, whilst calphostin C treatment showed a slight reduction in WNV replication, treatment with chelerythrine significantly reduced it, as measured by either the release of viral RNA to the culture medium or the quantification of dsRNA intermediates. These differences could be probably related to their different mechanism of action and specificity for PKC isoforms. Calphostin C interacts with the regulatory N-terminal domain of PKCs by competing for the binding site of DAG and phorbol esters, but not for Ca^2+^, while chelerythrine acts on the conserved catalytic C-terminal region [45]. Therefore, calphostin C is a more specific inhibitor for classical and novel PKCs and less specific for atypical ones, while chelerythrine can similarly inhibit all PKC isoforms. In this sense, the lack of inhibition produced by sotrastaurine and enzastaurin point to atypical isoforms as a specific target for WNV infection.

The role of PKCs has been studied in a wide variety of viruses. PKC inhibition of viral infection has already been reported in human parainfluenza viruses [46], Rift valley fever virus [47], herpes simplex virus-1 [48], respiratory syncytial virus [49], and human herpesvirus 8 [50], among others. However, published results for flavivirus infections are controversial. PKC phosphorylation of the C viral protein has been described as the key regulator of apoptosis induction during infection [25], and inhibition of all PKC isoforms affected WNV entry into mosquito cells [26]. Conversely, DENV replication was enhanced after in vitro inhibition with bisindlolylmaleinmide I (Bis I), a specific chemical inhibitor of classical and novel PKCs [17]. In our study, both calphostin C and chelerythrine decreased the amount of RNA released to the culture medium and of dsRNA intermediates formation, even though the observed reduction was significantly higher when chelerythrine was used. Thus, the specific relationships that occur between PKC isoforms and multiplication of viruses must be carefully addressed before future design of antiviral drugs to combat diseases that affect human and animal health. Nonetheless, the results point that some PKC inhibitors (i.e., chelerythrine) could become potential antiviral candidates for WNV, as well as for other related flaviviruses.
